# Hyperpigmentation Due to Cyclosporine Therapy

**DOI:** 10.7759/cureus.4072

**Published:** 2019-02-14

**Authors:** Ajay N Sharma, Allison S Dobry, Kenneth Linden

**Affiliations:** 1 Dermatology, University of California, Irvine, USA

**Keywords:** cyclosporine, hyperpigmentation, pigmentation, prurigo nodularis

## Abstract

For a myriad of immune disorders, cyclosporine has demonstrated marked efficacy in relieving clinical symptoms and reversing pathological developments. We present a case of hyperpigmentation induced by cyclosporine therapy used to treat prurigo nodularis, an extremely rare adverse effect of cyclosporine that has been reported only once, to our knowledge, in the dermatologic literature. After four months of cyclosporine therapy, our patient developed noticeable hyperpigmentation on the dorsal hands and feet and to a lesser degree on her arms and legs. Prior research has discovered a dose-dependent decrease in tyrosinase activity and pigment formation in cultured melanocytes due to cyclosporine – an effect opposite to what was observed in our case. Thus, further study into this relationship is necessary. In essence, physicians should be aware of unwanted cutaneous changes after the initiation of cyclosporine therapy and may want to counsel patients about the importance of ultraviolet (UV) radiation protection.

## Introduction

For over three decades, cyclosporine has been a core component of immunosuppression in both immune dysregulatory disorders and organ transplantation. For immune disorders involving ophthalmologic, dermatologic, hematologic, gastroenterologic, neurologic, or musculoskeletal systems, cyclosporine has demonstrated marked efficacy in relieving clinical symptoms and reversing pathological developments [1]. Additionally, after the drug’s implementation in transplantation medicine, rates of acute rejection and one-year graft survival have improved dramatically, although five-year survival rates have been disappointing [2]. Due to its myriad adverse side effects from its upstream inhibition of the immune system, cyclosporine is often reserved for refractory disease. We present a case of a patient with hyperpigmentation induced by cyclosporine therapy used to treat prurigo nodularis, an extremely rare adverse effect of cyclosporine that has been reported only once, to our knowledge, in the dermatologic literature.

## Case presentation

A 42-year-old Vietnamese woman with a history of chronic hepatitis B presented to the clinic for a widespread pruritic rash for the past two years. The rash was characterized by numerous 2-15 mm excoriated, indurated, hyperpigmented erythematous papules and plaques distributed on the bilateral arms and feet, trunk, and back. Punch biopsies from each arm demonstrated prurigo nodularis. Initial treatment of these lesions was twice daily clobetasol ointment.

A return to the clinic, one and a half months late, revealed an increased number of similar lesions and persistent pruritus. Given the lack of response to topical therapy, the patient was initiated on oral cyclosporine A at 100 mg twice a day dosing. A complete blood count, complete metabolic panel, lipid panel, uric acid level, and magnesium level were ordered at the time of first cyclosporine prescription. All laboratory results returned within normal limits.

**Figure 1:**
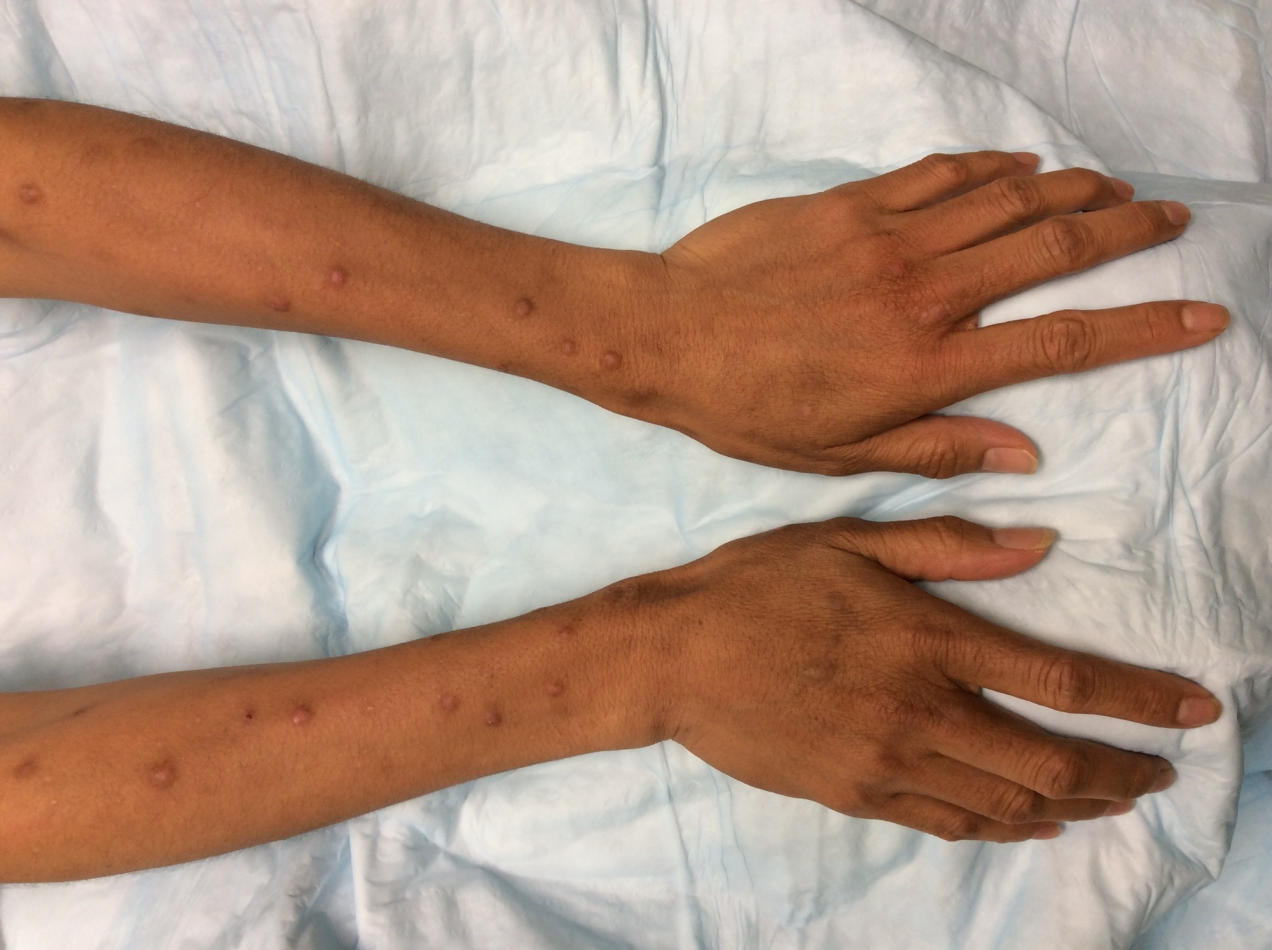
Hyperpigmentation of distal forearms and dorsal hands

The patient had good response to cyclosporine therapy, and after four months after cyclosporine initiation, her lesions were nearly all healed with the resolution of her pruritus. However, the patient noted that both she and her family felt her skin was becoming extremely tan and dry. Physical examination at that time was significant for marked hyperpigmentation mostly on the dorsal hands and feet and to a lesser degree on her arms and legs (Figure 1).

Thorough patient history revealed no sun exposure more than normal, no extended length of time exposing the dorsal hands or feet (i.e., wearing sandals), with family and friends commenting on the hyperpigmentation in these areas. Despite the changes in skin color, continuation of 100 mg cyclosporine was recommended due to continued efficacious therapeutic response for her prurigo nodularis. The patient was advised to use topical emollients (CeraVe; Coria Laboratories, Aliso Viejo, CA) and use sun protection while outside.

## Discussion

Cyclosporine has proven to be efficacious for a wide range of immunoregulatory disorders and solid organ transplant immunosuppression. A lipophilic cyclic polypeptide, cyclosporine exerts its therapeutic effect through the inhibition of calcineurin. This action results in the reversible inhibition of various cytokines’ transcription, including interleukin-2, and subsequent suppression of T helper lymphocyte activation [1-2].

Cyclosporine is available in oral, intravenous, and ophthalmic formulations. Bioavailability of the oral form is poor, averaging around 30% (range of 5%-70%) [1]. The drug is converted to less active metabolites by enzymes of the hepatic cytochrome P-450 enzyme superfamily, necessitating careful observation of potential drug interactions in the setting of polypharmacy, as well as monitoring of drug levels in the setting of hepatic dysfunction [3]. Various topical treatments (e.g., corticosteroids, calcipotriol) are often co-administered with cyclosporine to improve clinical efficacy, as in our patient [4].

In the dermatologic setting, cyclosporine has a role in the treatment of a host of pathologies including, but not limited to psoriasis, atopic dermatitis, pyoderma gangrenosum, chronic urticaria, Behçet disease, dermatomyositis, pemphigus vulgaris, epidermolysis bullosa acquisita, photodermatoses, lichen planus, and scleroderma [4]. With regards to cyclosporine’s role in prurigo nodularis, results have been positive in reducing the intensity of pruritus and frequency of new lesions [5-6]. Recommendations for oral dosing for prurigo nodularis consist of 3.5-4 mg/kg/day for 24-36 weeks. Clinical improvement can be observed as early as two weeks after beginning treatment [7]. The nodules classically seen in prurigo nodularis can be described as firm, pruritic, and hyperkeratotic, ranging from 2 mm – 2 cm in diameter. The trunk and extensor surfaces of the limbs are most commonly affected while the face and palms are less so, though no part of the body is immune [8]. Lesions are typically arranged linearly and the skin between lesions can be normal or at times xerotic [7]. Given the extent of disease and monomorphic appearance of all visible nodules, this case was a very atypical presentation of prurigo nodularis.

Drug-induced photosensitivity and hyperpigmentation are frequent developments from therapeutic agents, representing at least 8% of reported cutaneous adverse events from drugs [9]. Common drugs that alter pigmentation include nonsteroidal anti-inflammatory drugs, antimalarials, amiodarone, cytotoxic drugs, tetracyclines, azoles, and psychotropic drugs [10]. Potential photosensitizing medications include the aforementioned drug classes, along with hydrochlorothiazides, angiotensin-converting enzyme inhibitors, angiotensin receptor blockers, and some chemotherapeutic agents [9]. Cyclosporine has not been consistently listed as a photosensitizing drug. Diagnosis of drug-induced cutaneous changes relies primarily on the history of drug intake and appearance of eruption, though phototesting and photopatch testing are useful complementary tools. In this case, the patient was not taking any medications other than those prescribed for her prurigo nodularis.

The potentially adverse effects of cyclosporine are varied. The most severe concerns during treatment are hypertension and renal dysfunction, but dose-dependent hypertrichosis, gingival hyperplasia, and gastrointestinal upset are seen more commonly [1,11]. This case demonstrates an increase in the pigmentation of our patient’s dorsal hands, dorsal feet, arms, and legs. A review of the literature has revealed very few reported instances of cyclosporine-induced hyperpigmentation, although this development has been noted as early as 1989 (Table 1).

**Table 1:** Reported cases of patients with cyclosporine-induced hyperpigmentation PMID: PubMed Identifier

Reported cases of patients with cyclosporine-induced hyperpigmentation					
Authors (Year)	PMID	Patient Characteristics	Cyclosporine dosing	Locations of hyperpigmentation	Outcome of hyperpigmentation
Brady, Wing (1989) [12]	2502741	- 51-year-old white man - Received cadaveric renal transplant	-10 mg/kg initiated -Steady reduction over time	- Neck - Axilla - Hands	- Resolution after discontinuation of cyclosporine
Ozkaya-Bayazit et al. (2000) [13]	10972102	- 38-year-old white woman with acute myeloid leukemia	- Chemotherapy: Cytarabine, daunorubicin, etoposide, cyclosporine (2.5 mg/kg)	-Face - Occluded Skin	- No resolution described
Euvrard et al. (2001) [14]	11369903	- 145 children - Received renal, liver, or heart transplants	- Triple immunosuppressive therapy: prednisone, azathioprine, cyclosporine (unspecified dosages)	- 10 patients (6.9%) experienced diffuse hyperpigmentation -More pronounced on the upper trunk, arms	- Spontaneous resolution in four patients - No resolution after discontinuation of cyclosporine in one patient
Ardalan, Shoja (2009) [15]	19765472	- 24-year-old woman - Received allogenic renal transplant	- Triple immunosuppressive therapy: prednisolone, mycophenolate mofetil, cyclosporine (unspecified dosages)	- Forehead - Palmar creases	- Spontaneous resolution after several months
Muller et al. (2011) [16]	21781081	- 63-year-old man with Rowell syndrome	- 100 mg/day; reduction to 50 mg/day after 14 days	- Back	- Spontaneous resolution after four weeks

These previously reported patients who developed cyclosporine-induced hyperpigmentation were prescribed the medication for a severe, life-threatening condition: organ transplant acceptance, leukemia, and systemic autoimmune illness. Compared to our patient, no other case described hyperpigmentation observed on the feet, with the most commonly affected areas seen on the hands and face. Additionally, cyclosporine was most often used as part of a multiple pharmaceutical regimen, while it was employed only as monotherapy in our patient.

An in vitro study on the effect of cyclosporine on melanogenesis discovered a dose-dependent decrease in tyrosinase activity and pigment formation in cultured melanocytes – an effect opposite to what would be expected from clinical presentation [17]. Thus, increases in skin pigmentation observed post-cyclosporine therapy initiation are likely due to an indirect effect of the drug on melanocytes.

## Conclusions

Many of the adverse side effects of cyclosporine are well studied and often reported, including nephrotoxicity, hypertension, and gingival hyperplasia. As described in this report, cyclosporine-induced hyperpigmentation is a rare side effect of a common medication used in refractory dermatologic conditions as well as numerous other medical disorders. The pathomechanism of this potential adverse effect is unclear, but it is likely cyclosporine affects the development of melanocytes indirectly. Further study into this relationship is necessary; nevertheless, physicians should be aware of unwanted cutaneous changes after the initiation of cyclosporine therapy and may want to counsel patients about the importance of ultraviolet (UV) radiation protection.
